# Exome-Wide Analysis of the DiscovEHR Cohort Reveals Novel Candidate Pharmacogenomic Variants for Clinical Pharmacogenomics

**DOI:** 10.3390/genes11050561

**Published:** 2020-05-18

**Authors:** Maria-Theodora Pandi, Marc S. Williams, Peter van der Spek, Maria Koromina, George P. Patrinos

**Affiliations:** 1 Department of Pharmacy, School of Health Sciences, University of Patras, GR 26504 Patras, Greece; pha2665@upnet.gr (M.-T.P.); mkoromina@upnet.gr (M.K.); 2 Bioinformatics Unit, Department of Pathology, Faculty of Medicine and Health Sciences, Erasmus University Medical Center, 3015 GD Rotterdam, The Netherlands; p.vanderspek@erasmusmc.nl; 3 Geisinger, Danville, PA 7822, USA; mswilliams1@geisinger.edu; 4 Zayed Center of Health Sciences, United Arab Emirates University, Al-Ain, UAE; 5 Department of Pathology, College of Medicine and Health Sciences, United Arab Emirates University, Al-Ain, UAE

**Keywords:** Pharmacogenomics, PGx variants, allele distribution, gnomAD, PharmGKB

## Abstract

Recent advances in next-generation sequencing technology have led to the production of an unprecedented volume of genomic data, thus further advancing our understanding of the role of genetic variation in clinical pharmacogenomics. In the present study, we used whole exome sequencing data from 50,726 participants, as derived from the DiscovEHR cohort, to identify pharmacogenomic variants of potential clinical relevance, according to their occurrence within the PharmGKB database. We further assessed the distribution of the identified rare and common pharmacogenomics variants amongst different GnomAD subpopulations. Overall, our findings show that the use of publicly available sequence data, such as the DiscovEHR dataset and GnomAD, provides an opportunity for a deeper understanding of genetic variation in pharmacogenes with direct implications in clinical pharmacogenomics.

## 1. Introduction

The DiscovEHR cohort is the result of the collaboration between Geisinger (GHS) and the Regeneron Genetics Center. It is comprised of samples of GHS patients, who consented to participate in the Geisinger MyCode Community Health initiative [1,2]. Protein-coding regions (exome) of 18,852 genes in 50,726 DiscovEHR participants were sequenced at the Regeneron Genetics Center. The high-throughput sequencing data combined with deidentified longitudinal electronic health records (EHR) and other demographic details are used for genetic research purposes. With the term ‘deidentified,’ we refer to data from EHR records encrypted in order to prevent someone’s personal identity from being revealed. Additionally, the genetic data from DiscovEHR have been successfully used by GHS to detect causative variants associated with a variety of diseases, such as hereditary breast and ovarian cancer, familial hypercholesterolemia, Lynch syndrome, cardiomyopathy and many others that, once confirmed in a clinical laboratory, are returned to participants as part of clinical care [3,4].

Previous studies have focused on highlighting the importance of next-generation sequencing to support the integration of pharmacogenomics into clinical practice [5]. One of the first studies that demonstrated the value of next-generation sequencing in pharmacogenomics (PGx) was that of Mizzi et al. (2014), who performed bioinformatics analysis of whole-genome sequencing data from 482 unrelated individuals, thus leading to the identification of 408,964 variants in 231 pharmacogenes, of which 16,487 were novel. Further *in silico* analyses indicated that 1012 of the novel pharmacogene-related variants had the potential to abolish protein function [6].

In another study by Mizzi et al. (2016), significant inter-population pharmacogenomic biomarker allele frequency differences were observed within 7 clinically actionable pharmacogenomic biomarkers in 7 European populations, thus affecting drug efficacy and/or toxicity of 51 medication treatments [7]. Moreover, exploitation of whole genome sequencing (WGS) data in the Estonian biobank revealed 41 (10 of which were novel) loss-of-function and 567 (134 novel) missense variants in 64 very important pharmacogenes [8]. Interestingly, most of the identified variants were characterized by very rare frequencies below 0.05%. Overall, Tasa et al. (2019) demonstrated that population-based WGS-coupled EHRs are a useful tool for biomarker discovery [8].

Undoubtedly, there is sufficient scientific evidence supporting the contribution of rare variants in various complex and common diseases [9]. More precisely, deleterious alleles are likely to be rare owing to the evolutionary purifying selection [10]. However, little is known about the distribution and frequency of variants within clinically relevant pharmacogenes in different populations, especially for variants for which pharmacogenomics guidelines from the Clinical Pharmacogenetics Implementation Consortium (CPIC), PharmGΚB and the Dutch Pharmacogenetics Working Group (DPWG) exist.

In addition, large population-wide studies have led to the observation of a great number of rare, population-specific single nucleotide variations (SNVs) in protein coding genes, enriched in potentially deleterious changes, as a result of the rapid population growth, combined with weak purifying selection [11]. These findings were subsequently reproduced in a series of studies [12,13,14]. Furthermore, pharmacogenes, and more specifically genes whose products are involved primarily in pharmacokinetics, through weaker evolutionary selection, result in an abundance of Loss of Function (LoF) variants [15].

In this study, we assess common and rare pharmacogenomics (PGx) variants within the predominantly European Caucasian ancestry DiscovEHR cohort, whilst comparing our results with 501 PGx variants found in the 1000 Genomes dataset phase 3 (1kG-p3 dataset) [16], as well as with information from PharmGKB-CPIC gene-specific tables and with data from different gnomAD populations.

## 2. Materials and Methods

### 2.1. Study Population

We used freely available allele frequency data from whole-exome sequencing of 50,726 adult participants comprising the DiscovEHR cohort (http://discovehrshare.com). Extensive description of the DiscovEHR cohort is provided elsewhere [2].

### 2.2. Variant Annotation

The project-level VCF file, including all variants reported in the DiscovEHR cohort, was retrieved from the DiscovEHRshare website bcftools (version 1.6) and was used in order to extract variants located in the 231 Drug Metabolizing Enzymes and Transporters (DMET) pharmacogenes of interest (Table S1). Moreover, the project-level VCF file included only high-quality variants (i.e., PASS filter for all included variants). Variants were annotated using the Variant Effect Predictor (VEP, version for GRCh37) [17], keeping for each variant only the first entry in the transcripts field, since this transcript maintains the maximum annotation levels in all available annotation fields. Information for the minor allele frequencies (MAFs) of the assessed variants was retrieved from VEP and included gnomAD (version r2.1, exomes only) allele frequencies. Overall, 60,892 variants were processed and the results were filtered in order to retain only those corresponding to variants within DMET genes (54,318). Of these, 159 variants within DMET genes were annotated by VEP as ‘polymorphic_pseudogene’ and were excluded, thus the total number of variants kept for further analysis was 54,159.

Variants were classified based on their variant consequence by using the Sequence Ontology (SO) terms [18], as presented in ‘Consequence’ column in VEP’s output (GRCh37, 2020). Moreover, ‘loss-of-function’ (‘LoF’) variants were characterized with the following SO terms: ‘splice acceptor variant,’ ‘splice donor variant,’ ‘stop gained,’ ‘frameshift variant,’ ‘start lost’ and ‘stop lost’ [2,19]. We used the term ‘novel’ to describe variants that have not been reported in the Ensembl Variation database. In line with this VEP annotation, variants were also classified according to their predicted functional impact as ‘HIGH,’ ‘MODERATE,’ ‘LOW’ and ‘MODIFIER,’ which denotes the probability of the variants harboring a disruptive or non-disruptive effect on the protein.

### 2.3. Analysis of Annotated Variants

The entire analysis of the output file produced from VEP, after the initial filtering with the help of bcftools, was performed using a custom script in R programming language (available upon request). Variants were binned in five discrete MAF categories based on the following criteria: common (MAF >= 0.10 or MAF >= 10%), intermediate (0.05 =< MAF < 0.10 or 5% =< MAF < 10%), low frequency (0.01 =< MAF < 0.05 or 1% =< MAF < 5%), rare (0.001 <= MAF < 0.01 or 0.1% =< MAF < 1%) and ultra-rare (MAF < 0.001 or MAF < 0.1%). Subsequently, we compared the MAF distribution of rare and common PGx variants from the DiscovEHR cohort with the MAF distribution of these variants within different gnomAD subpopulations. In addition, we investigated the presence of overlaps between the identified PGx variants and 501 PharmGKB variants, shared across 26 populations, in the 1000 Genomes Project [16]. 

As a final step, we examined the potential of these variants to affect the function of the corresponding protein products, thus potentially leading to altered drug response. This analysis was performed according to the predictions of six *in silico* tools, as extracted from VEP direct plugins (CADD, PolyPhen 2.2.2, SIFT 5.2.2) or VEP’s plugin for dbNSFP’s 3.5a version (REVEL, MutPred, MutationAssessor). Variants were classified as damaging if at least 5 of 6 conditions were met: CADD_phred score equal or higher than 30, Polyphen prediction as ‘probably_damaging,’ SIFT prediction as ‘deleterious,’ REVEL score equals or higher than 0.85, MutPred score equals or higher than 0.75, MutationAssessor_pred prediction as ‘functional impact high (H).’ 

## 3. Results

### 3.1. Composition of Protein-Coding PGx-Related Variation in 50,726 Exomes

**Figure 1:**
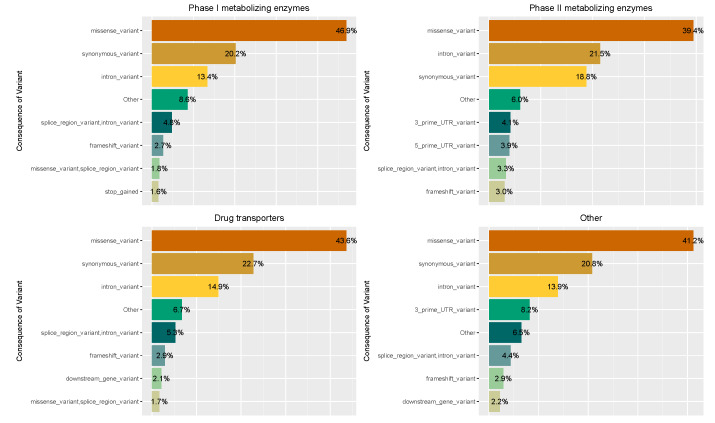
Percentage of the distribution of the VEP variant consequences of the identified pharmacogenomics (PGx) variants in 50,726 DiscovEHR exomes. 231 DMET genes were grouped in 4 categories: Phase I metabolizing enzymes, Phase II metabolizing enzymes, Transporters and Others. Abbreviations: DMET, Drug Metabolizing Enzymes and Transporters; VEP, Variant Effect Predictor.

Initially, we examined the most common VEP consequences allocated in each of the four main PGx gene families (Phase I and II metabolizing enzymes, Transporters and Others) [20] (Figure 1). As demonstrated in Figure 1, the three most frequently occurring categories, across all PGx gene families, are ‘missense’ (39.4–46.9%), ‘synonymous’ (20.2–22.7%) and ‘intronic’ (13.4–18.8%) variants. This is not unusual though, since whole exome sequencing usually captures intronic variants proximal to exons. Regarding variants characterized as ‘frameshift,’ we observed that they occur at a low frequency (2.7–3%) within the four main PGx gene families. Moreover, variants classified as ‘splice_region_variant’ were found within all assessed PGx gene families with their frequencies ranging from 3.3% to 5.3%. When it comes to the other less frequently occurring VEP consequences, the ‘stop gain’ variants range between 1.4% and 1.8% amongst the 4 pharmacogene families, ‘stop lost’ variants between 0.04% to 0.09% and ‘start lost’ variants are found in percentages ranging from 0.05% (in Other) and 0.25% (in Phase II enzymes).

**Figure 2:**
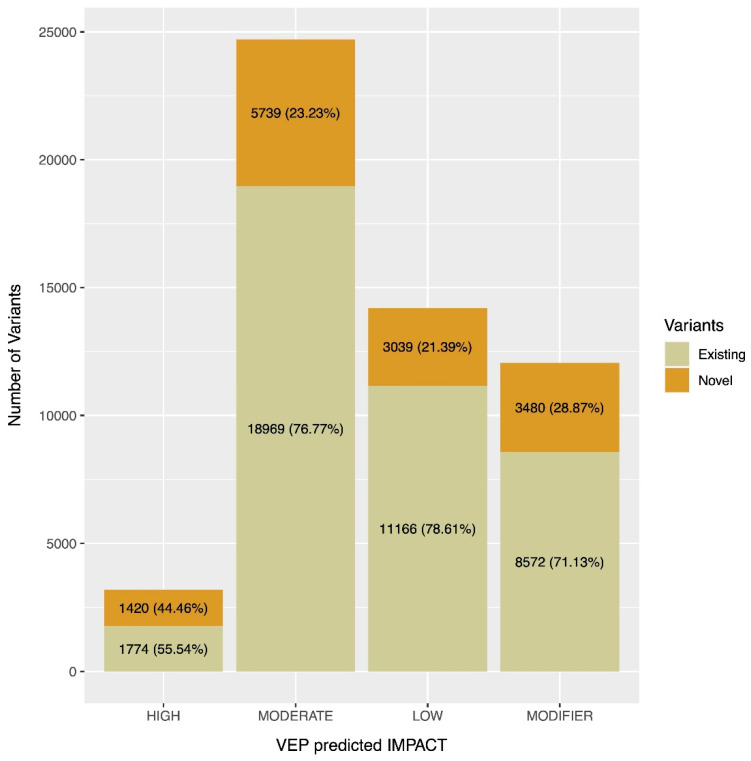
Number of novel and previously known PGx variants within 50,726 DiscovEHR exomes binned in 4 categories according to their VEP impact (‘HIGH,’ ‘MODERATE,’ ‘LOW,’ ‘MODIFIER’). The percentages of novel and existing variants within each VEP impact category are also provided. Abbreviations: PGx, pharmacogenomics.

Then, we assessed the number of novel and previously known PGx variants according to their VEP predicted impact (Figure 2). Interestingly, we observed that variants classified as ‘HIGH’ impact are characterized by a roughly equal distribution of novel and previously known variants. In contrast, the percentage of novel variants is lower compared to the previously known variation in the rest VEP impact categories (‘MODERATE,’ ‘LOW,’ ‘MODIFIER’).

### 3.2. Distribution of the Frequencies of the Identified PGx Variants within Different gnomAD Populations

**Figure 3:**
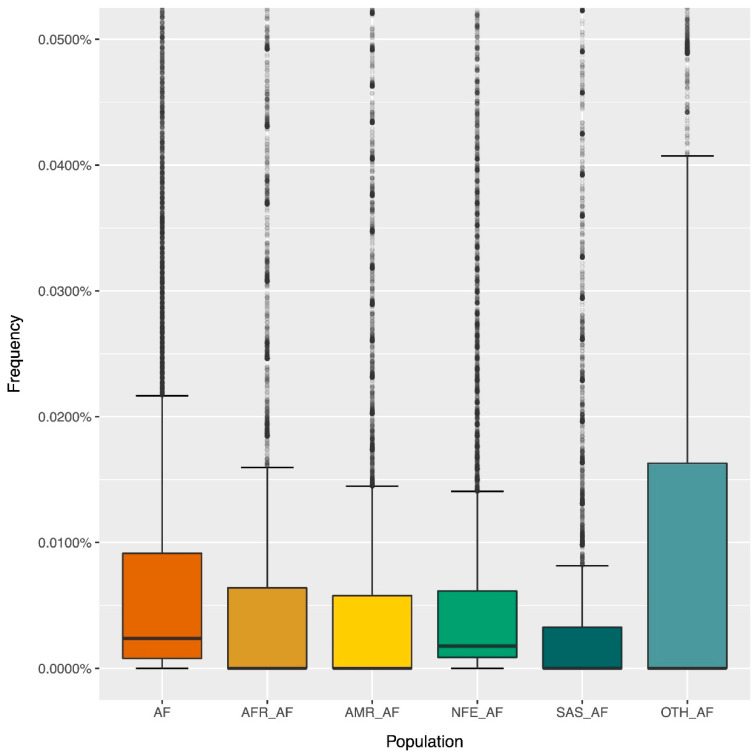
Boxplot of the MAFs (0–0.045%) of the PGx variants, identified within the DiscovEHR cohort, within various gnomAD populations. Abbreviations: MAF, minor allele frequency; PGx, pharmacogenomics; AF, combined gnomAD population; AFR_AF, African/African American; AMR_AF, Latino; NFE_AF, Non-Finnish European; SAS_AF, South Asian; OTH_AF, Other.

We subsequently examined the distribution of frequencies of the PGx variants, as identified within the DiscovEHR cohort, within different gnomAD populations (Figure 3). We specifically selected only those populations that reflect the population structure of the DiscovEHR cohort. More precisely, we assessed the following gnomAD populations: combined gnomAD population (AF), African/African American (AFR_AF), Latino (AMR_AF), Non-Finnish European (NFE_AF), South Asian (SAS_AF) and other (OTH_AF).

**Figure 4:**
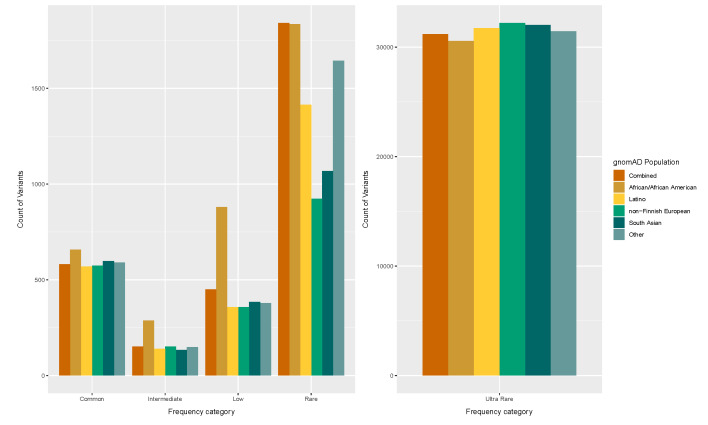
Variant counts in PGx genes, as identified in the DiscovEHR cohort, within various gnomAD populations. Variants were classified in the following categories: common (MAF >= 0.10 or MAF >= 10%), intermediate (0.05 =< MAF < 0.10 or 5% =< MAF < 10%), low frequency (0.01 =< MAF < 0.05 or 1% =< MAF < 5%), rare (0.001 <= MAF < 0.01 or 0.1% =< MAF < 1%), ultra-rare (MAF < 0.001 or MAF < 0.1%). Abbreviations: MAF, minor allele frequency; PGx, pharmacogenomics.

As shown in Figure 3, there are many outliers across the frequency range, whilst the main frequency distribution volume lies below 0.02% (Figure 3). The median frequency for the combined and the Non-Finnish European populations is higher than the other four presented populations, although it is still in low frequency levels (below 0.005%). Taken together, the frequency distributions for all presented gnomAD populations can be classified in the category of rare and ultra-rare variants. This observation is further supported in Figure 4, which demonstrates that ultra-rare variants (MAF < 0.1% or MAF = 0.001) are the prevailing frequency class across all the examined gnomAD populations.

### 3.3. Assessing the Protein Damaging Effect of Variants in the 231 DMET Genes within the DiscovEHR Cohort

Next, we examined the potentially protein damaging effect of the identified PGx variants. The filtering applied led to 804 variants spread across 66 pharmacogenes, with no single gene accumulating more than 4% of the variants. All 804 variants were characterized by a ‘MODERATE’ VEP Impact and their VEP consequence was either ‘missense_variant’ or ‘missense_variant,splice_region_variant.’ As shown in Table 1, most of the protein damaging variants are located in genes belonging to the PGx family of ‘Transporters.’ Although most of these variants are known, there are a significant number (22.4–33.1%) of novel protein damaging variants spread across all 4 assessed PGx gene families (Table 1). With regards to the corresponding population frequencies, we conclude that the vast majority of predicted damaging variants are considered as “ultra-rare,” while adequate information is available only for the combined population and the non-Finish European population (Figures S1 and S2). 

### 3.4. Assessing the Pharmacogenomics Clinical Relevance of the Identified PGx Variants 

We assessed the potential clinical relevance of our findings, by comparing our observations with the reported findings by Lakiotaki et al. (2017) [16]. Out of 501 reported PharmGKB variants found within the 1kG-p3 dataset shared across 26 populations, we found an overlap for 333 (approx. 66.46%) of those with the DiscovEHR cohort. The majority of the variants were located within genes encoding for Phase I metabolizing enzymes (*N* = 188 out of 333, 56.45%). Moreover, these variants were primarily characterized by a ‘MODERATE’ VEP impact (*N* = 158 out of 188, 84.04%), with ‘missense’ being the main VEP consequence (*N* = 154 out of 188, 81.91%). 

Table 2 and Table S2 show the distribution and the characterization of the shared PGx variants (*N* = 333), as retrieved from PharmGKB, between Lakiotaki et al. (2017) and the identified PGx variants from the DiscovEHR cohort [16]. Interestingly, variants classified as LoF (*N* = 13 out of 333, 4%) were found only within genes in Metabolizing Enzymes (Phase I and II) (Table S1). In addition, genes encoding phase I metabolizing enzymes encompass a wider variety of VEP consequences.

Comparison of our present findings with information retrieved from the gene-specific tables from the Pharmacogenomic Knowledge Base (PharmGKB, data accessed and curated on September 2019) and CPIC was also conducted. More precisely, we obtained information for 201 variants, for which protein function effect was determined either from experimental or clinical data (or both). As presented in Table 3, 91 of these variants were identified in our dataset, the majority of which (*N* = 77 out of 91, 84.62%) were located within the Phase I Metabolizing Enzymes gene family. The rest of these variants were located within the Phase II Metabolizing Enzymes and Transporters gene family (*N* = 9 and *N* = 5 out of 91, 9.9% and 5.5% respectively). 

In addition, ‘No function’ variants contain variation characterized by a variety of possible VEP impact values. The most prominent class is ‘MODERATE’ impact, however, ‘No function’ variants were the only ones with a ‘HIGH’ predicted impact. The remaining 3 functionality categories (‘Normal,’ ‘Possibly decreased,’ ‘Decreased’) are mostly ‘MODERATE’ impact variants (Table S3). Moreover, variants classified as loss of function and variants affecting the splicing lie within the ‘No function’ category, an observation supporting the overall agreement between VEP impact and protein function effect. On the other hand, missense variants seem to be related to a greater spectrum of predicted effects in protein function and are represented across more genes (Table 3). 

### 3.5. Assessing LoF PGx Variants within 50,726 Exomes

Analysis of the 50,726 exomes from the DiscovEHR cohort led to the identification of 3,194 LoF variants within 231 DMET genes. We observed an equal number of LoF variants identified within genes encoding for Phase I Metabolizing Enzymes and Transporters (*N* = 1,081 and *N* = 1,013 respectively). In contrast, Phase II Metabolizing Enzymes and Others were characterized by a lower number of LoF variants, almost half compared to the previously discussed PGx gene families (*N* = 581 and *N* = 519 respectively).

Moreover, we observed that 13 out of the 333 variants shared between the Lakiotaki et al. (2017) derived dataset and the DiscovEHR cohort were characterized as LoF [16]. Furthermore, 9 out of 91 shared PGx variants from the PharmGKB gene specific tables belonged to the LoF category, with 8 having a ‘No function’ protein effect and 1 leading to a ‘Decreased function’ protein product.

## 4. Discussion

### 4.1. Rare PGx Variation within the DiscovEHR Cohort

In this study, we demonstrated how we can expand knowledge about PGx variants by utilizing publicly available genetic data. More precisely, the results from this study demonstrate that a population from a single hospital system can be used to identify rare and ultra-rare pharmacogenomic-related variants with the potential for clinical effect on drug response. 

Exome-wide rare and common variant analysis within DMET genes in the DiscovEHR cohort led to the identification of 54,159 variants. By assessing the pharmacogenomics variation on 231 DMET genes, we came across a high number of rare (0.001 <= MAF < 0.01 or 0.1% =< MAF < 1%) and ultra-rare (MAF < 0.001 or MAF < 0.1%) variants. In addition, when assessing the MAF distribution of the identified PGx variants across different gnomAD populations, it was observed that the median allele frequency value for all gnomAD populations was low (median MAF below 0.01%). These observations further support the notion that the majority of the pharmacogenomic variation is rare [21]. 

In line with the findings by Dewey et al. (2016), who assessed all functional variants within the DiscovEHR cohort, we also found that the majority of the PGx variants are SNVs (*N* = 51,212), whilst insertion/deletion variants are found in lower numbers (*N* = 2,947) [2].

Furthermore, by examining the VEP consequences of the identified PGx variants, categorized in four main pharmacogene families (Phase I drug metabolizing enzymes, Phase II drug metabolizing enzymes, Transporters and Others), we observed that the majority of these were characterized as missense, synonymous and intronic (in total *N* = 43,208 of 54,159, 79.77%). Interestingly, variants characterized as LoF were found with lower occurrence frequencies within the DiscovEHR cohort compared to the other categories (i.e., missense, synonymous, intronic) (Figure 1). Although most of the identified pharmacogenomics variation was characterized as known, we still identified a high number of novel variants (*N* = 13,678 out of 54,159, 25.25%). Of note, almost half of the variants having a ‘HIGH’ impact were characterized as novel (*N* = 1,420 out of 3,194, 44.46%). The high number of LoF variants (i.e., ‘HIGH’ impact) is not surprising, given the size of the examined population (50,726), which allows the identification of such variants. Moreover, as shown previously, pharmacogenes are facing less strict evolutionary constrains, thus leading to an accumulation of LoF variants [15], a finding which further supports our results. 

### 4.2. Identifying Ultra-Rare Damaging PGx Variants within the DiscovEHR Cohort

The potentially protein damaging effect of the PGx variants was assessed by combining the scores and predictions from 6 different *in silico* tools. Unsurprisingly, most ‘predicted as damaging’ variants were also ultra-rare (frequency <0.01%). Interestingly, the frequency of the potentially damaging variants is slightly higher in Non-Finnish Europeans compared to all the other assessed populations, but it is still low (<0.005%). This could be explained by the demographic data of the DiscovEHR cohort (publicly available), which denote that the DiscovEHR cohort is primarily of non-Finnish Northern European descent. Another interesting observation is that there were a significant absolute number and proportion of ‘novel’ damaging variants (*N* = 245 of 804, 30.47%). 

In line with the present findings, Dewey et al. (2016) demonstrated that the largest proportion of the identified variants were non-synonymous variants with an allele frequency lower than 1%. Herein, we also show that a significant percentage of the identified PGx variation within DMET genes (27.47%) is also composed from ultra-rare (according to the combined gnomAD population), non-synonymous variants (i.e., missense) (*N* = 14,875 out of 54,159). Interestingly, no LoF variant was identified in the ‘ultra-rare’ frequency category.

The present findings are also in line with previous studies indicating that rare missense and LoF variants within pharmacogenes can determine interindividual differences in drug response. Kozyra and colleagues (2017) assessed the contribution of rare genetic variants in the 1KG and Exome Sequencing Project (ESP) and showed that the majority of variants within pharmacogenes were rare and nonsynonymous, whilst approximately 30%–40% of functional variability in pharmacogenes was attributed to rare variants [22]. In another study, the contribution of known and novel pharmacogenomics variants (including rare missense and LoF variants) within the two versions of gnomAD (i.e., v2 and v3) was assessed. Similar to our findings, it was shown that novel LoF and missense variants within DPWG pharmacogenes occurred with a MAF less than 0.1% [23]. Overall, findings from these studies combined with our findings further showcase that novel pharmacogenomics variants contribute to the significant variability in the distribution of PGx variants within different populations.

### 4.3. Towards Clinical Pharmacogenomics

When comparing with information retrieved from gene-specific tables of PharmGKB-CPIC, we identified 91 variants shared between those and the DiscovEHR cohort. With regards to ‘No function’ variants, we found a concordance between the VEP impact prediction, which is associated with the VEP consequence and the protein function effect, since ‘No function’ is the only class containing ‘HIGH’ impact variation. However, the remaining genomic changes, including these that lead to protein products with decreased function, are mainly characterized as having a ‘MODERATE’ impact.

Furthermore, we showed that 333 out of 501 PharmGKB variants, shared across 26 populations in 1000Genomes, were also identified in the DiscovEHR cohort as well. Interestingly, variants classified as ‘HIGH’ impact (i.e., loss of function variants) were identified exclusively within genes encoding for drug metabolizing enzymes Phase I and II. 

To further highlight the potential clinical utility of our results, we assessed for an overlap of our findings with pharmacogenes harboring a ‘level A CPIC guideline’ annotation. We found that 11 out of 212 DMET genes from the DiscovEHR cohort lie within this annotation category [*DPYD, CYP2C19, CYP2C9, SLCO1B1, VKORC1, CYP4F2, CYP2B6, CYP2D6, TPMT, CYP3A5, G6PD*]. These exact genes are also identified in the gene dataset (originally *N* = 38), which describes the 501 PGx variants found in the 1kG-p3 dataset from Lakiotaki et al. (2017) [16]. Finally, we observed that 9 out of the 11 genes from the PharmGKB gene specific tables have a ‘level A CPIC guideline’ annotation [*DPYD, CYP2C19, CYP2C9, SLCO1B1, CYP4F2, CYP2B6, CYP2D6, TPMT, CYP3A5*]. 

One of the major caveats of the present study lies within the fact that several pharmacogenetically-relevant variants are usually not covered using commercially available whole-exome capturing kits (e.g. CYP2C19*17, CYP3A5*3, VKORC1*2). As this study is based on whole-exome sequencing data, we have an increased probability of covering and thus assessing the majority of the pharmacogenetically-relevant variants.

Our study though has some limitations that aim to be addressed in future studies. Firstly, *in silico* prediction scores may not directly correlate with clinical effect, therefore using EHR data to study drug effectiveness and adverse events to further study variants is an interesting future direction. However, this approach could be problematic with ultra-rare variants, given the small numbers of patients and the variable exposure to medications predicted to be impacted by the protein alterations; nevertheless, this approach could produce interesting results for common pharmacogenomics variants.

In addition to this, future studies should further characterise the potential splicing effect of intronic variants as well, even quite far from the consensus splicing site, by implementing either whole genome sequencing or whole pharmacogene sequencing. Moreover, as denoted above, this study focused only on investigating coding and proximal intronic SNPs and small indels. Therefore, of particular interest for future studies are also large structural variants, such as copy number variants (CNVs), which were not investigated in the present study, since their detection requires different computational approaches.

## 5. Conclusions

In this study, we integrated NGS data from a well-characterized cohort of 50,000 individuals (DiscovEHR) with publicly available PGx data to investigate the potential clinical utility of known and novel PGx variants. This approach led to the identification and characterisation of DMET pharmacogenomics variants not only within the DiscovEHR cohort but also within different subpopulations from the largest genomics database to date (i.e., gnomAD). We also showed that the use of data from a large and clinically well-characterized cohort enables the identification of variants with potential clinical pharmacogenomics relevance. Moreover, we identified a large number of novel and ultra-rare protein-damaging variants within a variety of gnomAD subpopulations, thus demonstrating that novel and rare missense and LoF PGx variants contribute to the functional variability within DMET pharmacogenes. We envisage integrating the available genotype, phenotype and clinical data related in order to directly associate protein-damaging variants with clinical variable outcomes, thus promoting the integration of pharmacogenomics into the daily clinic practice.

## Figures and Tables

**Table 1:** Count of predicted protein damaging variants in four main PGx gene families according to their VEP consequences and their characterization as novel or known. Abbreviations: VEP, Variant Effect Predictor.

Pharmacogene Family	VEP Consequence	Novel/Previously Known
Phase I Metabolizing Enzymes	missense_variant: 214 missense_variant,splice_region_variant: 10	Novel: 69 Known: 155
Phase II Metabolizing Enzymes	missense_variant: 80 missense_variant,splice_region_variant: 5	Novel: 19 Known: 66
Transporters	missense_variant: 369 missense_variant,splice_region_variant: 9	Novel: 125 Previously known: 253
Others	missense_variant: 113 missense_variant,splice_region_variant: 4	Novel: 32 Previously known: 85

**Table 2:** Count of PGx variants per pharmacogene family and VEP impact within the shared PharmGKB variants between Lakiotaki et al. (2017) [16] and DiscovEHR cohort. Abbreviations: ENZ I, Phase I metabolizing enzymes; ENZ II, Phase II metabolizing enzymes.

Pharmacogene Category	N	GENES	IMPACT
ENZ I	188	*CYP4B1, DPYD, CYP2C19, CYP2C9, CYP2C8, CYP2E1, CYP1A1, CYP1A2, CYP4F2, CYP2A6, CYP2B6, CYP2A13, CYP2F1, CYP2S1, CYP1B1, CYP2D6, CYP39A1, CYP3A5, CYP3A7, CYP3A4, CYP3A43*	HIGH: 11 LOW: 10 MODERATE: 158 MODIFIER: 9
ENZ II	115	*SULT2A1, SULT1C2, UGT1A8, UGT1A10, UGT1A6, COMT, UGT2B15, TPMT, NAT1, NAT2*	HIGH: 2 LOW: 18 MODERATE: 54 MODIFIER: 41
TRANSPORTERS	22	*ABCC2, SLCO1B1, SLC22A1, ABCB1*	HIGH: 0 LOW: 5 MODERATE: 17 MODIFIER: 0
OTHERS	8	*CDA, VKORC1, G6PD*	HIGH: 0 LOW: 2 MODERATE: 5 MODIFIER: 1

**Table 3:** Distribution of the 91 variants, which were found both in the DiscovEHR dataset and the gene-specific tables from PharmGKB, based on their VEP consequence based on Sequence Ontology terms. The distribution is shown on a gene level and on a pharmacogene (PGx gene) family level.

Consequence	Number of Variants per Gene	Number of Variants per Functionality
frameshift_variant	*CYP2D6*: 2 *CYP3A5*: 1	No: 3
frameshift_variant,splice_region_variant	*CYP2C9*: 1	No: 1
inframe_deletion,splice_region_variant	*CYP2D6*: 1	Decreased: 1
intron_variant	*CYP2D6*: 2 *DPYD*: 1 *UGT1A6*: 2	Normal: 1 Decreased: 3 No: 1
missense_variant	*CYP2B6*: 4 *CYP2C19*: 3 *CYP2C9*: 7 *CYP2D6*: 2 *CYP4F2*: 1 *DPYD*:43 *SLCO1B1*: 5 *TPMT*: 4	Normal: 36 Possibly Decreased: 7 Decreased: 9 No: 17
missense_variant,splice_region_variant	*DPYD*:1	Normal: 1
splice_acceptor_variant	*CYP2D6*: 1 *TPMT*: 2	No: 3
splice_donor_variant	*DPYD*: 1	No: 1
splice_region_variant, intron_variant	*DPYD*: 1	Normal: 1
splice_region_variant, synonymous_variant	*DPYD*: 1	Normal: 1
start_lost	*TPMT*: 1	No: 1
synonymous_variant	*CYP3A5*: 1 *DPYD*: 3	Normal: 3 No: 1

## Supplementary Materials

The following are available online at https://www.mdpi.com/2073-4425/11/5/561/s1, Figure S1: Count of protein damaging missense variants based on their frequency category: low frequency (0.01 =< MAF < 0.05), rare (0.001 <= MAF < 0.01), ultra-rare (MAF < 0.001); Figure S2: Boxplot of the MAFs (0–0.04%) of the protein damaging missense PGx variants, identified within the DiscovEHR cohort, within various gnomAD populations. Table S1: Presentation of the 231 pharmacogenes according to their HGNC symbol (taken from Arbitrio M, Di Martino MT, Scionti F, et al. DMET™ (Drug Metabolism Enzymes and Transporters): a pharmacogenomic platform for precision medicine. Table S2: Count of shared PharmGKB variants between Lakiotaki et al. (2017) and DiscovEHR cohort according to the pharmacogene family and the VEP consequence based on Sequence Ontology terms. Table S3: Distribution of the 91 variants, which were found both in the DiscovEHR dataset and the gene-specific tables from PharmGKB, based on the VEP impact prediction and the protein function information as retrieved from PharmGKB.
