# Supplementary material for: Exome-Wide Analysis of the DiscovEHR Cohort Reveals Novel Candidate Pharmacogenomic Variants for Clinical Pharmacogenomics
Source: Genes (Basel). 2020 May 18;11(5):561. doi: 10.3390/genes11050561 (PMC7290308; doi:10.3390/genes11050561)
Supplement: Supplementary file 1 [file genes-11-00561-s001.zip › genes-767868-supplementary.pdf]

Supplemental Information

Exome-wide genome analysis of the DiscovEHR cohort reveals novel candidate pharmacogenomic variants for clinical pharmacogenomics

Maria-Theodora Pandi [1,2], Marc S. Williams [3], Peter van der Spek [2], Maria Koromina [1], George P. Patrinos [1,2,4,5, *]

[1] Department of Pharmacy, School of Health Sciences, University of Patras, Patras, Greece;

[2] Erasmus University Medical Center, Faculty of Medicine and Health Sciences, Department of Pathology, Bioinformatics Unit, Rotterdam, the Netherlands;

[3] Geisinger, Danville, PA, USA

[4] United Arab Emirates University, Zayed Center of Health Sciences, Al-Ain, UAE;

[5] United Arab Emirates University, College of Medicine and Health Sciences, Department of Pathology, Al-Ain, UAE

Figure S1. Count of protein damaging missense variants based on their frequency category: low frequency (0.01 =< MAF < 0.05), rare (0.001 <= MAF < 0.01), ultra-rare (MAF < 0.001).

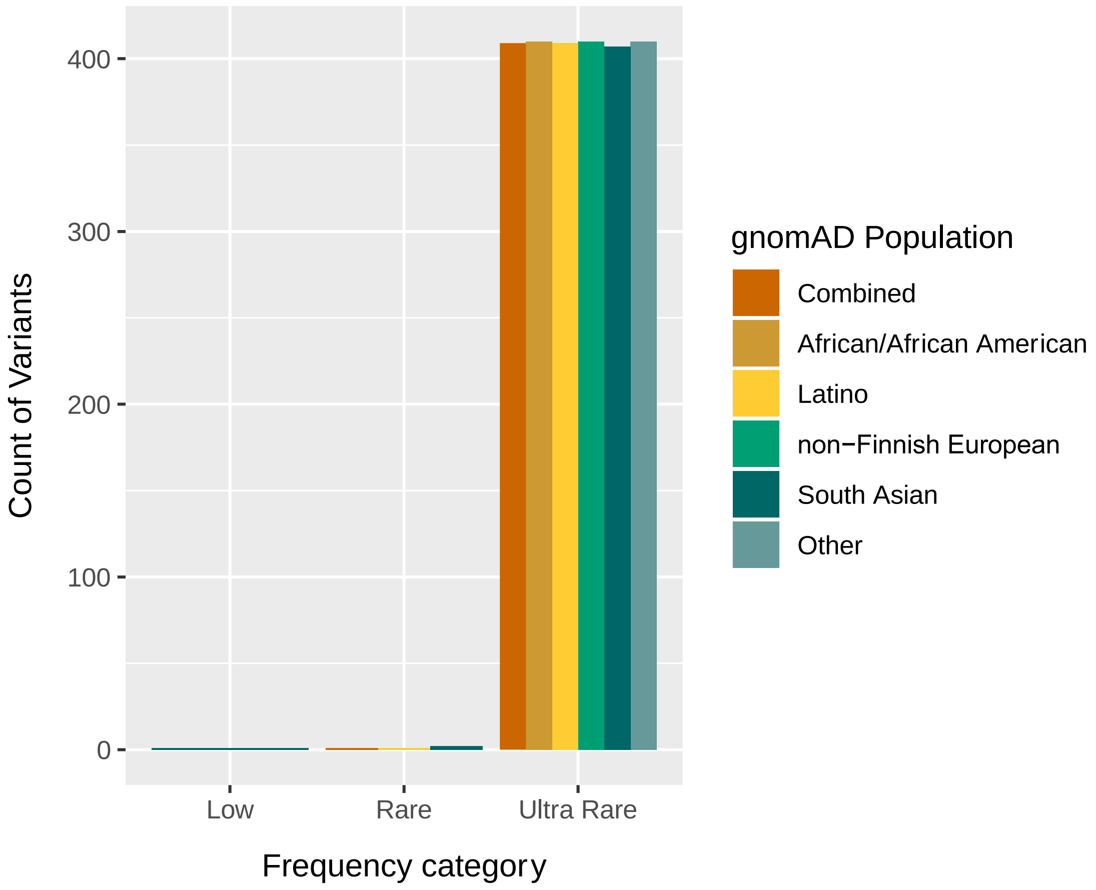

Figure S2. Boxplot of the MAFs (0% - 0.04%) of the protein damaging missense PGx variants, identified within the DiscovEHR cohort, within various gnomAD populations. Abbreviations: MAF, minor allele frequency; PGx, pharmacogenomics; AF, combined gnomAD population; AFR_AF, African/African American; AMR_AF, Latino; NFE_AF, Non-Finnish European; SAS_AF, South Asian; OTH_AF, Other.

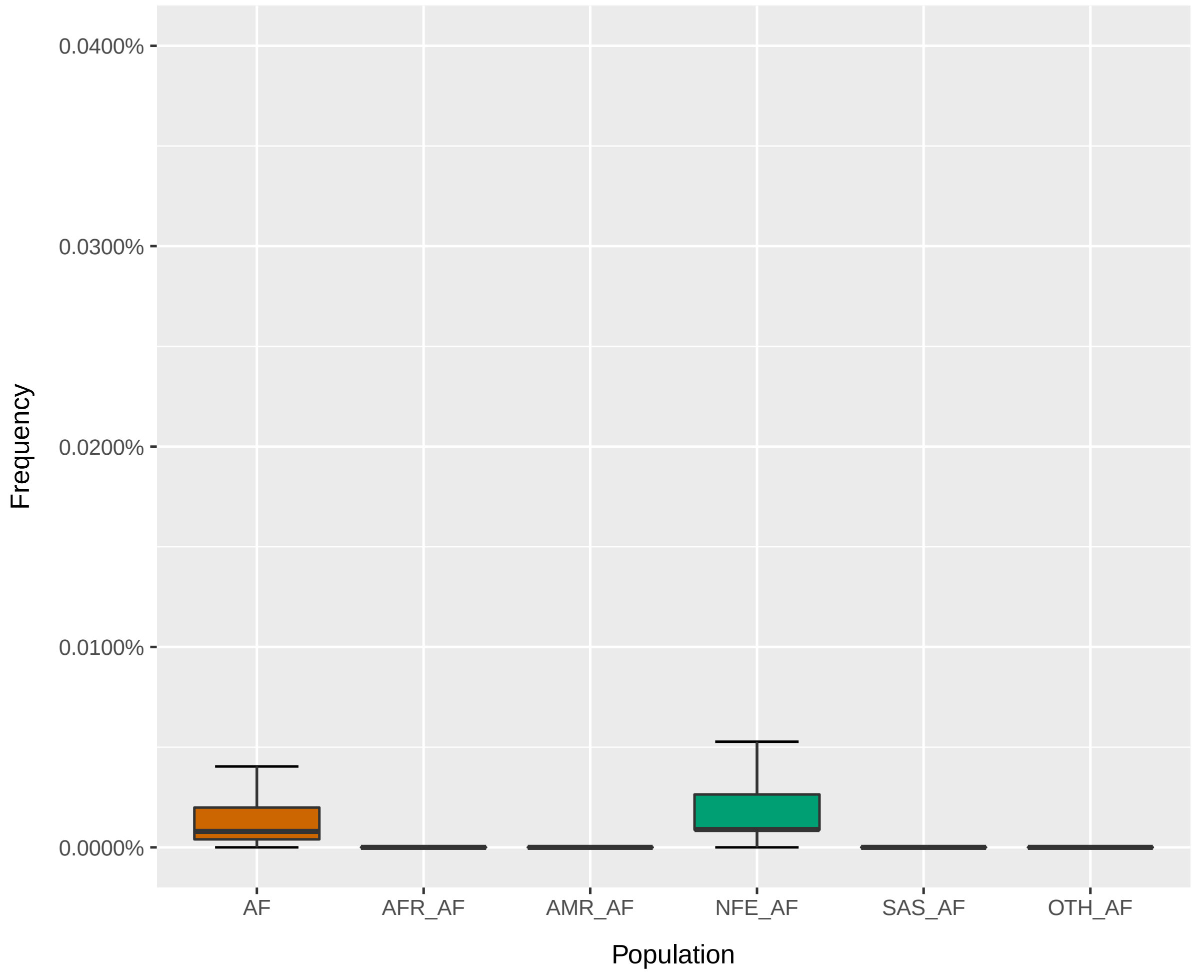

Table S1. Presentation of the 231 pharmacogenes according to their HGNC symbol (taken from Arbitrio M, Di Martino MT, Scionti F, et al. DMET™ (Drug Metabolism Enzymes and Transporters): a pharmacogenomic platform for precision medicine. Oncotarget. 2016;7(33):54028-54050. doi:10.18632/oncotarget.9927)

File name: Supplemental_Table_1.csv

Table S2. Count of shared PharmGKB variants between Lakiotaki et al. (2017) and DiscovEHR cohort according to the pharmacogene family and the VEP consequence based on Sequence Ontology terms. Abbreviations: ENZ I, Phase I metabolizing enzymes; ENZ II, Phase II metabolizing enzymes.

**Consequence**	**Number of variants per Pharmacogene category**
3_prime_UTR_variant	EnzII: 4
frameshift_variant	EnzI: 2
frameshift_variant,splice_region_variant	EnzI: 2
inframe_deletion,splice_region_variant	EnzI: 1
intron_variant	EnzI: 8<br>EnzII: 37<br>Other: 1
missense_variant	EnzI: 154<br>EnzII: 54<br>Transporters: 17<br>Other: 5
missense_variant,splice_region_variant	EnzI: 3
splice_acceptor_variant	EnzI: 1
splice_donor_variant	EnzI: 1
splice_donor_variant,coding_sequence_variant	EnzI: 1
start_lost	EnzI: 2
stop_gained	EnzI: 2<br>EnzII: 2
synonymous_variant	EnzI: 10<br>EnzII: 18<br>Transporters: 5<br>Other: 2
upstream_gene_variant	EnzI: 1

Table S3. Distribution of the 91 variants, which were found both in the DiscovEHR dataset and the gene-specific tables from PharmGKB, based on the VEP impact prediction and the protein function information as retrieved from PharmGKB. Normal function is coded as normal, possibly decreased protein function as possibly decreased, decreased protein function as decreased, and, absence of protein function or no function as no.

	**IMPACT**			
**Function**	HIGH	MODERATE	LOW	MODIFIER
Normal	0	37	5	1
Possibly Decreased	0	7	0	0
Decreased	0	10	0	3
No	9	17	1	1
